# Single low-dose rHuIL-12 safely triggers multilineage hematopoietic and immune-mediated effects

**DOI:** 10.1186/2162-3619-3-11

**Published:** 2014-04-11

**Authors:** Mamata S Gokhale, Vladimir Vainstein, Jamie Tom, Simmy Thomas, Chris E Lawrence, Zoya Gluzman-Poltorak, Nicholas Siebers, Lena A Basile

**Affiliations:** 1 Neumedicines Inc, 133 North Altadena Drive, Suite 310, 91107-7342 Pasadena, CA, USA; 2 Covance Clinical Research Unit, 3402 Kinsman Blvd, 53704 Madison, WI, USA

**Keywords:** IL-12, Human, Hematopoiesis, HSARS, Cancer

## Abstract

**Background:**

Recombinant human interleukin 12 (rHuIL-12) regulates hematopoiesis and cell-mediated immunity. Based on these hematopoietic and immunomodulatory activities, a recombinant human IL-12 (rHuIL-12) is now under development to address the unmet need for a medical countermeasure against the hematopoietic syndrome of the acute radiation syndrome (HSARS) that occurs in individuals exposed to lethal radiation, and also to serve as adjuvant therapy that could provide dual hematopoietic and immunotherapeutic benefits in patients with cancer receiving chemotherapy. We sought to demonstrate in healthy subjects the safety of rHuIL-12 at single, low doses that are appropriate for use as a medical countermeasure for humans exposed to lethal radiation and as an immunomodulatory anti-cancer agent.

**Methods:**

Two placebo-controlled, double-blinded studies assessed the safety, tolerability, pharmacokinetics and pharmacodynamics of rHuIL-12. The first-in-human (FIH) dose-escalation study randomized subjects to single subcutaneous injections of placebo or rHuIL-12 at 2, 5, 10, and 20 μg doses. Due to toxicity, dose was reduced to 15 μg and then to 12 μg. The phase 1b expansion study randomized subjects to the highest safe and well tolerated dose of 12 μg.

**Results:**

Thirty-two subjects were enrolled in the FIH study: 4 active and 2 placebo at rHuIL-12 doses of 2, 5, 10, 12, and 15 μg; 1 active and 1 placebo at 20 μg. Sixty subjects were enrolled in the expansion study: 48 active and 12 placebo at 12 μg dose of rHuIL-12. In both studies, the most common adverse events (AEs) related to rHuIL-12 were headache, dizziness, and chills. No immunogenicity was observed. Elimination of rHuIL-12 was biphasic, suggesting significant distribution into extravascular spaces. rHuIL-12 triggered transient changes in neutrophils, platelets, reticulocytes, lymphocytes, natural killer cells, and CD34^+^ hematopoietic progenitor cells, and induced increases in interferon-γ and C-X-C motif chemokine 10.

**Conclusion:**

A single low dose of rHuIl-12 administered subcutaneously can elicit hematological and immune-mediated effects without undue toxicity. The safety and the potent multilineage hematopoietic/immunologic effects triggered by low-dose rHuIL-12 support the development of rHuIL-12 both as a radiation medical countermeasure and as adjuvant immunotherapy for cancer.

**Trial registration:**

ClinicalTrials.gov: NCT01742221

## Introduction

Interleukin-12 (IL-12), a heterodimeric cytokine with p40 and p35 subunits, is well-known for its pleiotropic effects. Several *in vitro* studies in the early-mid nineties reported that IL-12 is capable of stimulating hematopoiesis synergistically with other cytokines [1-5]. The hematopoiesis-promoting activity of IL-12 was suggested to be due to a direct action on bone marrow stem cells, thereby promoting the proliferation and/or differentiation of hematopoietic progenitor cells [3,6,7]. In addition to these hematopoietic effects of IL-12, the cytokine has demonstrated potent immunomodulatory effects. For example, IL-12 has been shown to play an essential role in the interaction between the innate and adaptive arms of immunity [6]. IL-12 has also been shown to enhance cytolytic activity of macrophages, T cells, and natural killer (NK) cells, and to stimulate the differentiation of naïve T helper (TH) cells into TH1 cells [8-12]. These immunomodulatory effects of IL-12 have been widely studied in pre-clinical models of diseases such as cancer [13,14], viral and parasitic infections [15,16], and allergy [17]. The importance of immune-mediated effects of IL-12 has been documented clinically by the discovery that immunodeficient patients experiencing recurrent infections harbor mutations in IL-12 or IL-12 receptor components [18,19]. IL-12 therapy also has been shown to restore resistance to infection after injury [20].

The effector mechanisms of IL-12 in innate resistance and adaptive immunity have been explored in various experimental tumor models, as reviewed by Colombo and Trinchieri [21]. Promising data obtained in preclinical models led to clinical trials in oncology patients with the hope that IL-12 could be a powerful anticancer therapeutic agent [21]. Indeed, immunomodulatory effects of IL-12 have been demonstrated in several human cancers [22-24]. However, the high doses and repeat-dosing regimens used in oncology trials generally resulted in excessive clinical toxicity that was accompanied by only a modest clinical response [21]. Various studies have implicated interferon gamma (IFN-γ), the hallmark of immune activation by IL-12, as an important mediator of antitumor activity [13,14,25,26]. However Gollob and colleagues found that induction of IFN-γ was markedly attenuated after repeat dosing of rHuIL-12 [25], indicating a tachyphylactic response. This inability to maintain IFN-γ release may have contributed to reduced tumor response rates in previous clinical trials of IL-12, where high doses and frequent administration regimens were used [21,26]. As these early phase trials did not meet expectations, further clinical development of IL-12 was not pursued. These historical data underscore the fact that anti-tumor potential of IL-12 has not been fully realized.

Based on its hematopoietic and immunomodulatory activities, a recombinant human IL-12 (rHuIL-12) is now under development to address the unmet need for a medical countermeasure (MCM) against the hematopoietic syndrome of the acute radiation syndrome (HSARS) that occurs in individuals exposed to lethal radiation, and also to serve as adjuvant therapy that could provide dual hematopoietic and immunotherapeutic benefits in patients with cancer receiving chemotherapy. These development paths are predicated on the use of single, low doses of IL-12, which had been shown to facilitate endogenous hematopoiesis in mice exposed to ionizing radiation [27]. The development program for rHuIL-12 as a medical countermeasure to lethal radiation exposure follows the FDA’s Animal Rule, where efficacy is determined in appropriate animal models and safety is demonstrated in humans [28]. To date, studies in irradiated non-human primates (NHP) and mice have demonstrated the ability of single, low doses of rHuIL-12 to induce recovery of hematopoietic tissue following radiation-induced bone marrow ablation and to decrease the incidence of hemorrhage and infections, all of which lead to a substantial increase in survival after exposure to lethal radiation [6]. These studies underscore the potential of a single, low dose of IL-12 to provide dual hematopoietic and immunologic benefits. Herein, we report the results of two clinical studies conducted in parallel to these animal efficacy studies to evaluate the safety and tolerability of single, low doses of rHuIL-12 in normal healthy subjects. The first-in-human (FIH) phase 1, dose-escalation study identified the maximum tolerated dose (MTD) of rHuIL-12 based on stringent toxicity criteria and the subsequent phase 1b expansion study demonstrated the safety of the rHuIL-12 at the MTD in a larger sample of healthy adults. These clinical safety studies support the development of rHuIL-12 as a stand-alone frontline radiation MCM. An additional important objective of these safety studies was to assess the safety of initiating development of rHuIL-12 as a multilineage hematopoietic and immunotherapeutic agent in patients with cancer who are receiving aggressive chemotherapy, where single doses of IL-12 are planned to be used once per chemotherapy cycle.

## Results

### Subject disposition

In the FIH dose-escalation study, 32 subjects were randomized: 21 subjects were randomized to rHuIL-12 (4 each at doses of 2, 5, 10, 12, and 15 μg and 1 the 20 μg dose) and 11 were randomized to placebo (2 each for doses of 2, 5, 10, 12, and 15 μg and 1 the 20 μg dose). All 32 subjects completed the study. Nineteen subjects (59%) were males and 13 (41%) were females; mean age was 28 years (range: 18 to 44 years). Twenty-six subjects (81%) were white, 2 (6%) were African American, 2 (6%) were Asian, and 2 (6%) were of other races.

In the subsequent Phase 1b expansion study, 60 subjects were randomized 4:1 to rHuIL-12 at the MTD identified in the FIH study (12 μg) or placebo; thus 48 subjects were treated with rHuIL-12 and 12 subjects were treated with placebo. Forty-nine subjects (82%) were males and eleven (18%) were females; mean age was 28 years (range: 18 to 45 years). Forty subjects (67%) were white, 13 (22%) were African American and 7 (11%) were of other races. Fifty-eight subjects completed the study. Two subjects withdrew (one on Day 16 and other on Day 22) for personal reasons unrelated to the study.

### Safety and tolerability

All 32 subjects in the FIH study and all 60 subjects in the phase 1b expansion study were analyzed for the primary endpoint.

#### *FIH study*

No serious AEs or AEs leading to discontinuation and no clinically significant changes in clinical chemistry laboratory evaluations, vital sign measurements, physical examinations, or 12-lead ECGs were observed in either study. All AEs were resolved or recovered by the end of the study. A summary of drug-related AEs in the FIH study is presented in Table 1. The most frequent AEs related to rHuIL-12 treatment were headache, dizziness, chills and injection site pain. All the AEs related to rHuIL-12, were mild (grade 1) to moderate (grade 2), except for three severe (grade 3) hematological AEs reported at the two highest doses. These included lymphopenia (absolute count of 400 lymphocytes/mm^3^) and thrombocytopenia (absolute counts of 109,000 to 118,000 platelets/mm^3^ over 4 days) in the single subject treated at the 20 μg dose level, and neutropenia (absolute counts of 700 to 1000 neutrophils/mm^3^ over 4 days) in 1 subject treated at the 15 μg dose level. The severe AEs started 3 or 4 days after dosing and continued for 3 to 4 days. The 2 events (grade 3 lymphopenia and grade 3 thrombocytopenia) occurring at the 20 μg dose level constituted dose-limiting toxicities (DLTs), and led to dose reduction of rHuIL-12 to 15 μg. Another DLT (grade 3 neutropenia) at 15 μg resulted in dose reduction of rHuIL-12 to 12 μg. None of the severe drug-related AEs required concomitant medication or other action. The maximum tolerated dose of 12 μg was further examined in the subsequent phase 1b expansion study.

**Table 1:** Number and percentage of subjects experiencing study drug related adverse events in the FIH study

**System organ class/preferred term**	**Placebo**	**rHuIL-12**	**rHuIL-12**	**rHuIL-12**	**rHuIL-12**	**rHuIL-12**	**rHuIL-12**
		**2 μg**	**5 μg**	**10 μg**	**12 μg**	**15 μg**	**20 μg**
	**(N = 11)**	**(N = 4)**	**(N = 4)**	**(N = 4)**	**(N = 4)**	**(N = 4)**	**(N = 1)**
	**n (%)**	**n (%)**	**n (%)**	**n (%)**	**n (%)**	**n (%)**	**n (%)**
Blood and Lymphatic System Disorders							
Leukopenia							1 (100%)
Lymphopenia	-	-	-	-	-	-	1 (100%)
Neutropenia	-	-	-	-	-	1 (25%)	
Thrombocytopenia	-	-	-	-	-	-	1 (100%)
Gastrointestinal Disorders							
Abdominal Distension	-	-	-	-	1 (25%)	-	-
Dyspepsia	-	-	-	-	1 (25%)	-	-
Nausea	-	-	-	-	-	-	1 (100%)
Paraesthesia Oral	-	-	-	-	1 (25%)	-	-
General Disorders and Administration Site Conditions							
Chills	-	-	-	-	1 (25%)	1 (25%)	1 (100%)
Fatigue	-	-	-	-	-	1 (25%)	-
Feeling Abnormal	-	1 (25%)	-	-	-	-	-
Feeling Hot	-	-	-	-	-	-	1 (100%)
Injection Site Erythema	2 (18%)		-	-	-	1 (25%)	1 (100%)
Injection Site Hemorrhage	1 (9%)						
Injection Site Pain	1 (9%)				1 (25%)	1 (25%)	
Injection Site Reaction	1 (9%)	-	-	-	1 (25%)	-	-
Local Swelling	-	-	-	-	1 (25%)	-	-
Malaise	-	-	-	-	-	-	1 (100%)
Pain	-	-	-	-	1 (25%)	-	-
Infections and Infestations							
Viral Infection	-	-	1 (25%)	1 (25%)	-	-	-
Tonsillitis	-	1 (25%)	-	-	-	-	-
Musculoskeletal and Connective Tissue Disorders							
Back Pain		-	-	-		1 (25%)	-
Myalgia	-	-	1 (25%)	-	-	1 (25%)	--
Musculoskeletal Pain	1 (9%)	-	-	-	-	-	-
Nervous System Disorder							
Headache	1 (9%)	-	-	-	3 (75%)	3 (75%)	1 (100%)
Dizziness		-	-		1 (25%)	1 (25%)	-
Hypoaesthesia	-	-	1 (25%)	-	-	-	-
Paraesthesia	-	-	-	-	1 (25%)	-	-
Presyncope		1 (25%)	-	-		-	-

*Abbreviations:N* total number of subjects in the dose group, *n (%)* number (%) of subjects who experienced each type of adverse event.

#### *Phase 1b expansion study*

A summary of drug-related AEs that occurred in ≥ 5% subjects in the expansion study is presented in Table 2. No subject treated with the single, 12 μg dose of rHuIL-12 used in this study experienced drug-related severe cytopenias, confirming the safety and tolerability observed in the FIH study. The most frequently reported rHuIL-12-related AEs were headache, dizziness, chills, fatigue, myalgia and back pain. All the AEs related to rHuIL-12 were mild or moderate except for episodes of severe chills and dizziness that occurred in the same subject beginning 11 hours after dosing; both events resolved over the next 5 hours, and neither required treatment or other action. Thus, data from the expansion study confirmed the overall safety and tolerability of rHuIL-12 observed in the FIH study, and expanded the size of the population treated safely at the 12 μg dose level.

**Table 2:** Study drug related adverse events occurring in ≥ 5% of Subjects in the Phase 1b expansion study

**System organ class (preferred term)**	**Placebo**	**rHuIL-12 12** **μg**
	**(N = 12)**	**(N = 48)**
Blood and Lymphatic System Disorders		
Lymphadenitis	0	6 (13%)
Thrombocytopenia	0	3 (6%)
Eye Disorders		
Photophobia	1 (8%)	0
Gastrointestinal Disorders		
Nausea	3 (25%)	7 (15%)
Vomiting	1 (8%)	2 (4%)
General Disorders and Administration Site Conditions		
Chills	1 (8%)	11 (23%)
Fatigue	1 (8%)	7 (15%)
Feeling Hot	3 (25%)	5 (10%)
Injection Site Erythema	0	5 (10%)
Injection Site Pain	1 (8%)	5 (10%)
Injection Site Reaction	0	4 (8%)
Pain	0	3 (6%)
Investigations		
Alanine Aminotransferase Increased	0	3 (6%)
Musculoskeletal and Connective Tissue Disorders		
Myalgia	1 (8%)	11 (23%)
Back Pain	0	10 (21%)
Arthralgia	0	3 (6%)
Musculoskeletal Stiffness	1 (8%)	2 (4%)
Neck Pain	1 (8%)	1 (2%)
Nervous System Disorders		
Headache	4 (33%)	26 (54%)
Dizziness	0	5 (10%)

*Abbreviations*: *N* total number of subjects in the dose group, *n (%)* number (%) of subjects who experienced each type of adverse event.

### Pharmacokinetics

**Figure 1:**
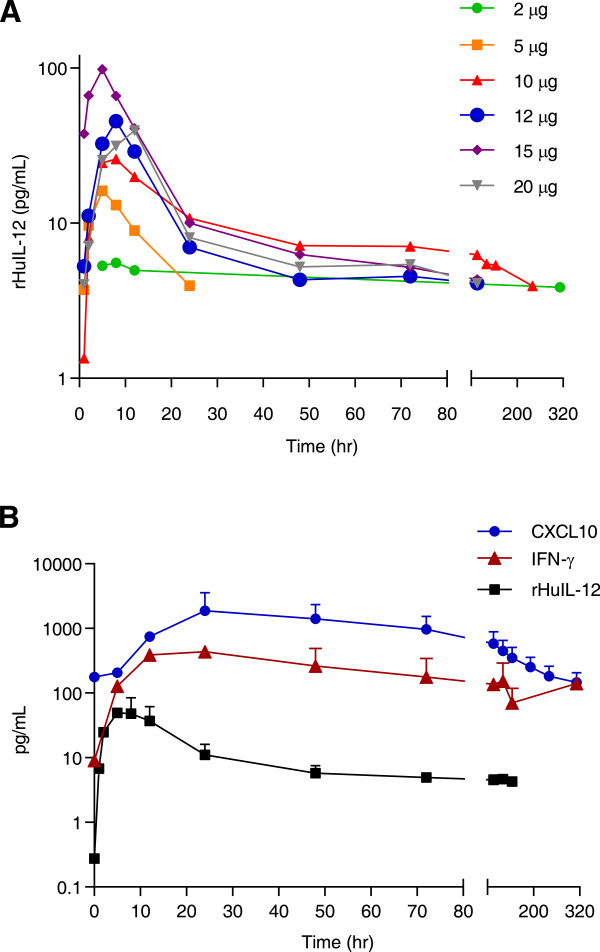
**Plasma concentration profiles of rHuIL-12 over time. A)** Plasma concentration vs. time for rHuIL-12 at 2, 5, 10, 12, 15 and 20 μg doses in the FIH study. **B)** Plasma concentration vs. time for rHuIL-12 (12 μg) and for IFN-γ and CXCL10 in subjects treated with rHuIL-12 at 12 μg in the phase 1b expansion study.

All subjects who received rHuIL-12 and had evaluable data were included in the pharmacokinetic (PK) analysis. Due to insufficient data in the terminal phase, exposure parameters requiring extrapolation, such as AUC_0-∞,_ elimination half life, volume of distribution and clearance, could not be reliably estimated in either study. The initial half life was calculated within the time frame of 8 to 24 hours after dosing while the terminal half-life was evaluated at later time points; the calculation for each subject required at least 3 descending data points beyond 24 hours after dosing. Plasma concentration vs. time profiles for rHuIL-12 are presented in Figure 1A (FIH study) and 1B (expansion study). Figure 1B also shows plasma concentration vs. time profiles for IFN-γ and C-X-C motif chemokine 10 (CXCL10; also known as IFN-γ-induced protein 10) in relation to rHuIL-12. These pharmacodynamic (PD) results are discussed with other PD results below.

#### *FIH study*

Mean PK parameters for rHuIL-12 after single SC injections in the FIH study are shown in Table 3. Sixteen of the 17 subjects who received rHuIL-12 (2 to 20 μg doses) had measureable systemic drug concentrations. The initial and terminal half-lives following an SC dose of rHuIL-12 could not be determined at the 2 and 5 μg doses due to inadequate data. There were no obvious trends in T_max_ with increasing doses of rHuIL-12. The C_max_ values generally increased with increasing dose, with the exception of the 20 μg cohort, where only 1 subject was dosed. Area-under-the-curve (AUC_0-t_) increased consistently as the dose increased from 2 to 15 μg. At 20 μg. there was a decrease in AUC_0-t_, based on the available single-subject data. Overall, increases in rHuIL-12 dose resulted in greater exposure. There were no gender differences.

**Table 3:** PK parameters for single dose rHuIL-12 in the FIH and Phase 1b expansion studies

	**PK Parameter**						
**Study**	**Dose**	**Statistic**	**Initial T**_**½**_	**Terminal T**_**½**_	**T**_**max**_	**C**_**max**_	**AUC **_**0-t**_
	**(μg)**		**(h)**	**(h)**	**(h)**	**(pg/mL)**	(h*pg/mL)
**FIH**							
	2	N	4	4	4	4	4
		Mean ± SD	NR	NR	8.00 (8.00 – 8.00)^a^	5.55 ± 1.34	45.28 ± 11.30
	5	N	4	4	4	4	4
		Mean ± SD	NR	NR	5.00 (2.00 - 8.00)^a^	16.52 ± 2.87	154.22 ± 56.41
	10	N	3	4	4	4	4
		Mean ± SD	11.8 ± 1.79	89.56 ± 57.61	8.00 (5.00 - 8.00)^a^	26.39 ± 3.90	1169.86 ± 513.05
	12	N	4	4	4	4	4
		Mean ± SD	9.23 ± 5.78	48.92 ± NR	8.00 (8.00 - 12.00)^a^	45.41 ± 43.46	790.74 ± 305.24
	15	N	4	4	4	4	4
		Mean ± SD	8.71 ± 6.54	90.19 ± 68.49	5.00 (5.00 - 8.00)^a^	96.81 ± 57.12	1436.67 ± 602.18
	20	N	1	1	1	1	1
		Mean ± SD	7.22	83.05	12.00^b^	39.50	970.58
**Expansion**							
	12	N	27	10	32	32	32
		Mean ± SD	8.74 ± 4.73	56.92 ± 42.20	8.00 (2.00 – 24.00)^a^	57.67 ± 49.84	1016.07 ± 612.97

*Abbreviations*: *N* number of subjects with sufficient data, *NR* not reported due to insufficient data, *N/A* not applicable, *SD* standard deviation.

^a^All data are means (±SD), except for T_max_, which is presented as median (range).

^b^Only 1 subject, range N/A.

#### *Phase 1b expansion study*

In the expansion study, a subset of 40 of the 60 enrolled subjects participated in the PK study. Of these, 32 were randomized to rHuIL-12 and 8 were randomized to placebo. Plasma rHuIl-12 was not detected in any of the 8 subjects who received placebo. All 32 subjects who received rHuIL-12 had measureable systemic concentrations after dosing, and endogenous rHuIL-12 was detected in 1 of the 32 subjects before treatment with rHuIL-12. Mean PK parameters are shown in Table 3. Systemic exposure of rHuIL-12 was variable, i.e., the coefficients of variance for C_max_, and AUC_0-t_ were 86%, 60% respectively. As shown in Figure 1B, the rHuIL-12 concentration versus time profile appeared to be biphasic, with an initial drop in plasma concentrations occurring around 8 hours after dosing and a terminal prolonged phase occurring at approximately 56 hours after dosing, suggesting significant distribution into the extravascular spaces. The slower terminal phase could be assessed only for 10 of the 32 subjects treated with rHuIL-12. There were no gender differences in rHuIL-12 exposure. Mean PK parameters from this study were comparable to those observed at the 12 μg SC dose level of rHuIL-12 in the FIH study.

### Immunogenicity

Plasma samples from all subjects in both studies were assayed for the presence of anti-drug antibodies to rHuIL-12. In both studies, samples from all subjects were tested at baseline (before dosing) and 28 days after dosing. In the Phase 1b expansion study, immunogenicity testing also was performed at day 45 in a subset of 34 subjects who consented to additional sampling. No confirmed anti-drug antibodies to rHuIL-12 were observed at the specified time points in any subjects in either treatment group in either study. Thus, there was no evidence that any of the PD or PK parameters for rHuIL-12 were confounded by the presence of anti-rHuIl-12 antibodies.

### Pharmacodynamics: hematological effects

As a part of the biological response to rHuIL-12, the hematological effects on various blood cell types were characterized over the course of the study. Neutrophils, platelets, lymphocytes, reticulocytes were examined in both studies, and specific lineages of lymphocytes and progenitor cells were examined in the Phase 1b expansion study only.

#### *Neutrophils, platelets, lymphocytes and reticulocytes*

##### 

**Figure 2:**
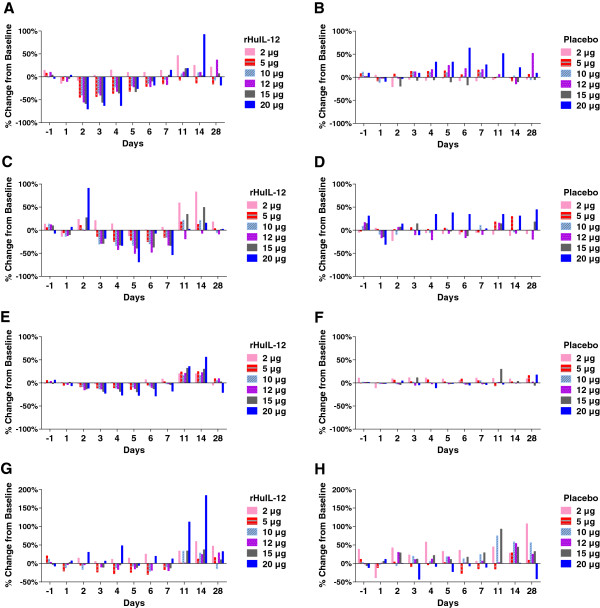
**Transient hematological changes with different rHuIL-12 doses and placebo in the FIH study.** The percentage changes from baseline count after treatment with rHuIL-12 (2, 5, 10, 12, 15, or 20 μg) or placebo are shown as follows: **A)** lymphocytes/rHuIL-12; **B)** lymphocytes/placebo; **C)** neutrophils/rHuIL-12; **D)** neutrophils/placebo; **E)** platelets/rHuIL-12; **F)** platelets/placebo; **G)** reticulocytes/rHuIL-12; **H)** reticulocytes/placebo.

**FIH study** All 32 subjects were monitored for hematological changes during the in-house stay (7 days) and at return visits on days 11, 14 and 28. Time-dependent, transitory changes in peripheral blood cell counts were seen in subjects treated with rHuIL-12 over the 28-day period, while counts remained more or less stable following placebo treatment. Figure 2 indicates the percentage change in lymphocyte, neutrophil, platelet, and reticulocyte counts relative to baseline in subjects treated with placebo or rHuIL-12 at the different dose levels. In rHuIL-12-treated subjects, lymphocytes and platelets decreased after day 1 and reached their respective nadirs between days 2 and 6; both types of cells then gradually increased, exceeding the baseline level at day 11 and returning to baseline by day 28. Neutrophils initially increased on day 2 and then decreased, reaching the nadir on day 5, and then followed the pattern observed for lymphocytes and platelets. Reticulocytes decreased after day 1, reached the nadir on day 6, and then followed the same pattern observed for lymphocytes, platelets, and neutrophils.

At the 20 μg dose of rHuIL-12, the nadirs for lymphocytes, neutrophils, and platelets were 70%, 69%, and 29% below baseline, respectively (based on data from one subject), which constituted DLTs per protocol criteria and led to a dose reduction from 20 μg to 15 μg, as described above. At the 15 μg dose of rHuIL-12, nadirs for lymphocytes, neutrophils, and platelets ranged from 53% to 69% below baseline, 33% to 64% below baseline, and 17% to 33% below baseline, respectively. A 64% decrease in neutrophils in one subject met the criteria for a DLT, as described above, and resulted in dose reduction to 12 μg. All DLTs were transitory.

##### 

**Figure 3:**
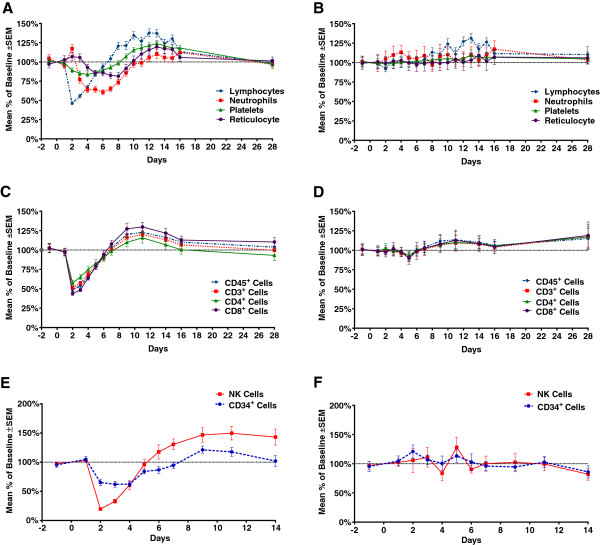
**Transient hematological changes with 12** **μg dose of rHuIL-12 or placebo in phase 1b expansion study.** Standard hematologic methods were used to determine cell counts at the indicated time points. The mean percentage of baseline count after treatment with rHuIL-12 (12 μg) or placebo are shown as follows: **A)** lymphocytes, neutrophils, platelets, and reticulocytes after treatment with rHuIL-12; **B)** lymphocytes, neutrophils, platelets, and reticulocytes after treatment with placebo; **C)** CD45+, CD3+, CD4+ and CD8+ cells after rHuIL-12; D) CD45+, CD3+, CD4+ and CD8+ cells after rHuIL-12; **D)** NK and CD34+ after placebo; **E)** NK and CD34+ cells after rHuIL-12; **F)** NK and CD34+ cells after placebo. NK cells were defined as CD45 + CD16 + CD56+ triple positive lymphocytes.

**Phase 1b expansion study** All subjects were monitored for hematological changes during the in-house stay for 16 days and at return visit on day 28. There was no difference in the absolute baseline counts for lymphocytes, neutrophils, and platelets between the treatment groups. These changes were similar to the transient decreases, recovery and increases seen at the 12 μg dose of rHuIL-12 in the FIH study. Figure 3 panel A describes mean percentage changes from baseline in lymphocytes, neutrophils, platelets, and reticulocytes after rHuIL-12 treatment. After dosing with rHuIL-12, treatment-related, transient decreases in lymphocytes and platelets were observed between days 2 and 8; both cell types returned to baseline by days 8 to 9, increased above baseline through day 16, and again returned to baseline by day 28. Similarly, neutrophils increased on day 2 and then decreased from day 3 to 9, returned to baseline by day 10 and stabilized by day 28. Reticulocytes increased on day 2, decreased from day 4 to 8, returned to baseline on Day 10, increased above baseline between days 10 and 16, and returned to baseline by day 28. None of these changes from baseline in any cell type were deemed to be clinically significant. Similar transient hematological changes were not seen after placebo treatment (Figure 3A panel B).

#### *Lymphocyte subpopulations and progenitor cells (expansion study only)*

In the expansion study, the transient hematological changes described in Figure 3 Panels A and B were further characterized using flow cytometry to detect specific lineages of blood cells such as CD3^+^, CD4^+^, CD8^+^ and CD45^+^ lymphocytes, NK cells, and CD34^+^ hematopoietic progenitor cells, as well as rHuIL-12Rβ2 positivity on NK and CD34^+^ progenitor cells, and mean fluorescence intensity of CD56 on NK cells.

As shown in Figure 3, Panel C, treatment-related decreases for CD3^+^, CD4^+^, CD8^+^, and CD45^+^ lymphocytes were observed from day 2 to 6 after dosing with 12 μg of rHuIL-12. Values for each lymphocyte subtype returned to baseline by day 7, which paralleled the changes in total lymphocytes (see Figure 3A), then transiently increased, with a peak around day 11, and again returned to baseline on day 28. The decrease from baseline at the nadir was between 40% and 55% and the increase from baseline at the peak was between 16% and 30%, depending on the cell type. Such changes were not seen with placebo treatment.

NK cells represented nearly 10% of the total peripheral blood cells, while the CD34^+^ progenitor cells accounted for only 0.1% (data not shown). Figure 3, Panel E, shows rHuIL-12 treatment-induced transient decreases in both NK and CD34+ cells. NK cells reached a nadir at 20% of the baseline level on day 2, and CD34+ cells reached a nadir at 62% of baseline on day 4. Levels returned to baseline by day 5 for NK cells and by day 7 for CD34^+^ progenitor cells. NK cells increased to baseline by day 5, exceeded baseline starting on day 6, peaked on day 11, and stabilized at a level 150% above baseline on day 14. CD34^+^ cells increased to baseline at day 7, exceeded baseline on day 9, and returned to the baseline level on day 14. Thus, NK cells recovered more rapidly than did CD34+ cells. With placebo treatment, both cell types fluctuated above and below the baseline, but changes were not comparable to those seen with rHuIL-12 treatment.

**Figure 4:**
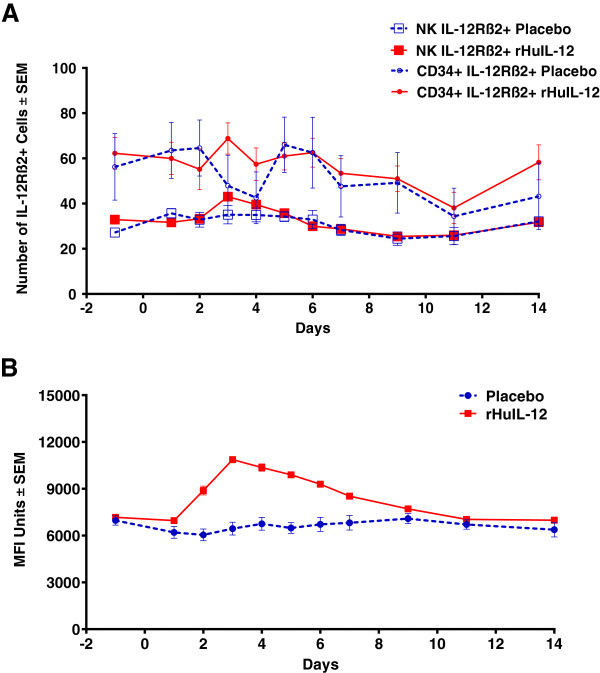
**Effect of rHuIL-12 or Placebo on rHuIL-12Rβ2 Positivity and CD56 Mean Fluorescence Intensity in the Phase 1b Expansion Study.** Flow cytometry was used to determine **A)** changes in the percentage of rHuIL-12Rβ2 positive NK and CD34+ cells in response to rHuIL-12 (12 μg) or placebo; and **B)** changes in CD56 mean fluorescence intensity on NK cells in response to dose of rHuIL-12 (12 μg) or placebo. NK cells were defined as CD45 + CD16 + CD56+ triple positive lymphocytes.

Cell surface expression of IL-12Rβ2, a unique subunit of the heterodimeric receptor for rHuIL-12, was studied to evaluate the effect of rHuIL-12 on the receptor positivity of NK and CD34^+^ progenitor cells. Over the course of the study, about 50% of the NK and CD34^+^ progenitor cells were IL-12Rβ2^+^, which represented approximately 5% and 0.05% of total peripheral blood cells, respectively. As shown in Figure 4A, rHuIL-12 treatment did not affect the number of IL-12Rβ2^+^ cells relative to that observed with placebo, although more fluctuations were seen in the number of receptor-positive CD34^+^ progenitor cells than in the number of receptor-positive NK cells.

Mean fluorescence intensity of CD56 on NK cells was increased with rHuIL-12 administration, peaking on day 3 and returning to baseline by day 11 (Figure 4B). No such changes were seen with placebo treatment. Overall, flow cytometric analysis showed that a single administration of rHuIL-12 to healthy subjects resulted in a rapid decreases in various types of peripheral blood cells followed by recovery at differing rates and extents.

### Pharmacodynamics: immunological effects

PD responses to rHuIL-12 were measured by quantifying levels of IFN-γ (both studies) as well as erythropoietin (EPO), interleukin-18 (IL-18) and CXCL10 (expansion study only).

#### *FIH study*

IFN-γ levels were not quantifiable (i.e., they were below the lower limit of quantification [LLOQ]) in response to 2 and 10 μg doses of rHuIL-12. At other doses of rHuIL-12, the T_max_ of IFN-γ (15 to 72 hours after rHuIL-12) was delayed relative to the T_max_ of rHuIL-12 itself (5 to 12 hours after dosing), which is indicative of a PD response (data not shown). However the exposure values for IFN-γ were quite variable and the composite data did not support a clear dose-PD or PK-PD relationship. Mean C_max_ values were 272 ± 176 pg/mL, 215 ± 148 pg/mL, 287 ± 181 pg/mL and 410 pg/mL respectively in response to 5, 12, 15 and 20 μg doses of rHuIL-12 and the mean AUC_0-t_ values were 13,962 ± 17,034 h*pg/mL, 13,984 ± 4473 h*pg/mL, 10,822 ± 6,994 h*pg/mL and 27,437 h*pg/mL respectively after 5, 12, 15 and 20 μg doses of rHuIL-12. The detection of systemic IFN-γ after rHuIL-12 dosing at 5 μg, but not at 10 μg was unexpected.

#### *Expansion study*

Endogenous IFN-γ was detected in 1 of the 32 subjects before treatment with rHuIL-12. A measureable IFN-γ response was observed 28 of the 32 subjects treated with rHuIL-12 (Figure 1B), but in none of the 8 subjects who received placebo (data not shown), indicating a positive PD response to rHuIL-12 treatment. The median IFN-γ T_max_ value (24 hours) was delayed in time relative to the median rHuIL-12 T_max_ value (8 hours; Figure 1B). Mean AUC_0-t_ value was 27,088 ± 22,380 h*pg/mL. There were no gender differences in IFN-γ response to rHuIL-12 (data not shown).

**Figure 5:**
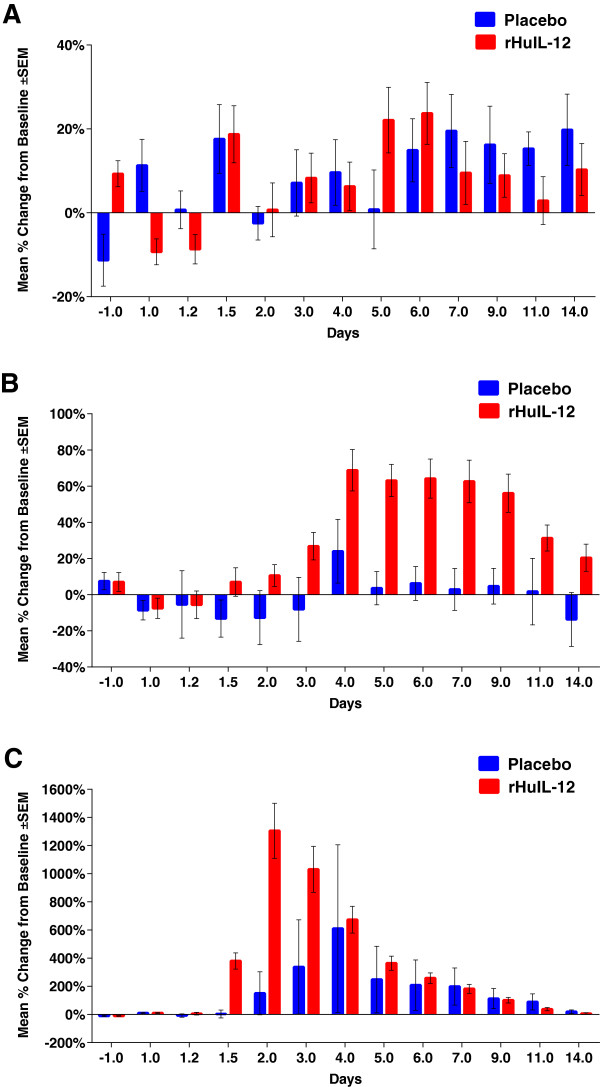
**Effect of rHuIL-12 or Placebo on EPO, IL-18 and CXCL10 levels in the Phase 1b expansion study.** EPO, IL-18, and CXCL10 were measured at the indicated time points using validated assays. The mean changes from baseline after treatment with rHuIL-12 (12 μg) or placebo are shown as follows: **A)** EPO; **B)** IL-18; **C)** CXCL10.

Endogenous EPO was detected prior to treatment with study drug in all 32 subjects. Over the 14 day period following SC injection of study drug, EPO levels fluctuated in both treatment groups, without any apparent response to rHuIL-12 treatment (Figure 5A).

Endogenous IL-18 also was detected prior to treatment with study drug, and, unlike EPO, showed a definite response to rHuIL-12 between 36 hours and 14 days after treatment with rHuIL-12 (Figure 5B). By comparison, IL-18 levels remained relatively close to baseline in the placebo-treated group (Figure 5B). Median T_max_ (107 hours) for IL-18 in the rHuIL-12 group was delayed relative to the median T_max_ for rHuIL-12 (8 hours; data not shown). Male subjects had a significantly higher (p ≤ 0.0002) IL-18 response than did females (data not shown).

Like EPO and IL-18, endogenous CXCL10 was detected prior to treatment with study drug. A PD response to rHuIL-12 was observed between 36 hours and 3 days after rHuIL-12 treatment, relative to placebo treatment (Figure 5C). The median CXCL10 T_max_ (24 hours) in subjects treated with rHuIL-12 was delayed relative to the median T_max_ for rHuIL-12 itself (8 hours; Figure 1B). In the placebo-treated group, only 2 of the 8 subjects showed a measurable CXCL10 response (data not shown), and the mean C_max_ was significantly lower than that of the rHuIL-12-treated group (1480.53 pg/mL with placebo vs. 2055.53 pg/mL with rHuIL-12; p < 0.03 by *T*-test on log transformed data). This difference may be confounded, however, by the extent and variability of endogenous levels observed in placebo-treated subjects (data not shown). There were no gender differences in the CXCL10 response to rHuIL-12 (data not shown).

## Discussion

The FIH and Phase 1b expansion studies reported here were designed to study the safety of rHuIL-12 when administered as a single SC dose of up to 20 μg in healthy subjects and to characterize the PK profile and hematological and immune-mediated effects of rHuIL-12. Characterization of IL-12’s effects in normal healthy subjects with intact immune systems is essential for further development of rHuIL-12 as a hematopoietic and immunotherapeutic agent for conditions such as HSARS or cancer.

The FIH study used a conservative range of single 2 to 20 μg doses, which are equivalent to 0.03 to 0.3 μg/kg in a 70 kg person. The SC route was chosen as it represents the route of choice for a front line medical radiation countermeasure and is also appropriate for cancer patients based on historical data showing that SC administration of IL-12 limited the toxic effects observed with intravenous treatment [29,30].

Per protocol, dose reductions were required in the event that one or more subject experienced a single Grade 3 change in clinical laboratory values, based on a stringent toxicity grading scale for healthy subjects [31]. At the 20 μg dose, the sentinel subject experienced Grade 3 thrombocytopenia and lymphopenia; thus, no additional subjects were dosed in this cohort. An additional cohort was enrolled at the reduced dose level of 15 μg, and one of 4 rHuIL-12-treated subjects experienced Grade 3 neutropenia. An additional cohort was therefore enrolled for treatment at an intermediary dose of 12 μg. As no DLTs were observed in this final cohort, the MTD was determined to be 12 μg. None of the drug-related, severe cytopenias observed in the FIH study resulted in any clinically significant manifestations and the absolute platelet and neutrophil counts were well above the levels which are considered predisposing to hemorrhagic or infectious episodes. No non-hematologic DLTs associated with rHuIL-12 occurred at any doses in the FIH study. The tolerability of the MTD identified in the FIH study (12 μg) was confirmed in the expansion study, as no clinically significant changes in vital signs, ECGs and physical examinations were observed in either study.

The most common AEs related to rHuIL-12 treatment in both studies were headache, dizziness, and chills. These AEs were expected and have been documented in previous studies as flu-like symptoms [10,30,32-34]. Incidence of fever was 39% in an earlier study of healthy volunteer [10] that used similar doses of IL-12, while it was 2% in our study. Thus, rHuIL-12 administered as single, SC doses up to 12 μg was found to be safe and well tolerated in healthy subjects in these studies.

Data from these studies showed that rHuIL-12 induced transient decreases in multiple peripheral blood cell counts, namely lymphocytes, neutrophils, platelets and reticulocytes as well as subpopulations of lymphocytes, i.e., CD3^+^, CD4^+^, and CD8^+^ cells, NK cells, and CD34^+^ hematopoietic progenitor cells. All of these cell populations subsequently recovered at different rates and resolved to their pre-dose baseline levels, or slightly above. The early onset and rapid reversibility of these changes indicate that they do not result from suppression of proliferation of hematopoietic precursors and progenitors in bone marrow, which otherwise would be expected to start later and last longer. These transient hematological changes can be explained on the basis of trafficking and redistribution of cells from the central blood compartment, e.g., neutrophil margination and exit into tissues, and lymphocyte redistribution to lymphoid organs. An additional explanation could be the delayed release of mature cells from the bone marrow post-mitotic compartments. The transient changes in neutrophil and lymphocyte counts observed in our studies have been reported by others [10,30,32,34,35]. However the rHuIL-12-induced transient changes in platelets and reticulocytes are novel findings in our study. We have not yet studied the underlying mechanism for these changes. However similar findings with respect to platelets have been reported with rHuIL-10, where a mild reversible decline in platelet counts was seen following 10 daily doses in healthy subjects [36]. Viability of the platelets was not affected in that study.

In addition to the parameters described above, safety also was assessed by testing for the development of anti-rHuIL-12 antibodies using a validated assay. Results showed no evidence of immunogenicity associated with rHuIL-12 in the FIH study (measured after 28 days) or the expansion study (measured after 45 days).

To our knowledge, this is the first report of a detailed PK analysis for rHuIL-12 in human subjects. The validated ELISA used to measure systemic concentrations of rHuIL-12 was sensitive enough to detect rHuIL-12 levels at all dose levels examined. There were no gender differences in rHuIL-12 exposure. Elimination of rHuIL-12 was biphasic with a short initial half-life of 8 to 24 hours measurable in all subjects and a prolonged terminal half-life > 50 hours measurable only in 47% subjects, possibly due to limitation of the rHuIL-12 assay. This terminal half- life possibly represents a slow return of rHuIL-12 to the central compartment from the tissues, and/or slow absorption from the SC space via the lymphatics and/or capillaries. Another explanation could be the secondary expression of endogenous IL-12 produced through a positive feedback loop [37]. In the FIH study, the rHuIL-12 exposure generally increased with increase in the dose. Overall, rHuIL-12 PK exposure in both studies was highly variable. The clearance and volume of distribution could not be reliably determined due to the lack of sufficient data in the terminal phase.

IL-12 is known to up-regulate adhesion molecules. *In vitro* studies have demonstrated that IL-12 is chemotactic to NK cells and stimulates adhesion of NK cells to endothelial cells and platelets via activation and increased expression of cell-cell adhesion molecules CD56 and CD11a [38,39]. The margination effect of lymphocytes and increased expression of adhesion molecules on NK cells was also demonstrated in cancer patients treated with IL-12 [35]. CD34 is also an important adhesion molecule required for T cells to enter lymph nodes [40,41]. In the expansion study, expression of CD56 levels as measured by mean fluorescence intensity increased after rHuIL-12 administration, while no such increase was seen in response to placebo. There also was an increase in CD34^+^ cells in response to rHuIL-12, but not with placebo. These observations provide a mechanistic explanation for the transient hematological changes. In addition, analysis of NK and CD34+ progenitor cells for IL-12Rβ2 positivity confirmed that these cells can be directly targeted by rHuIL-12. During the course of the study, relative percentages of IL-12Rβ2+ cells after rHuIL-12 treatment were similar to those observed after placebo treatment, suggesting that rHuIL-12 did not down regulate IL-12Rβ2 within these two cell populations.

Overall the hematological changes observed in both studies suggest that rHuIL-12 induces trafficking of peripheral blood cells from the vasculature to the tissues as a part of normal immune surveillance. Interestingly, the effect of rHuIL-12 on peripheral blood cells was multilineage, affecting all major blood cell types. To the best of our knowledge, this is the first example of a single cytokine that simultaneously induces a decline followed by recovery of lymphocytes, neutrophils, platelets, reticulocytes, as well as subpopulations of lymphocytes. We speculate that these changes are resulting from the activation and distribution of the cells into tissues. Definitive studies are needed to understand this phenomenon. Data relevant to our speculation have been reported before in nonclinical and clinical studies. Gately et al. [42] have reported a study where livers of IL-12 treated mice revealed focal mononuclear cell infiltrates. The livers contained increased numbers of NK cells, CD8+ T cells, and monocytes. Also the liver and splenic lymphoid cells from these IL-12-treated mice spontaneously secreted IFN-γ *in vitro,* suggesting that they had been induced by IL-12 to produce IFN-γ *in vivo*. This was indeed confirmed as IFN-γ levels were detected in the serum of IL-12-treated mice. In another murine study, Jackson et al. [43] reported that a single injection of recombinant murine IL-12 significantly reduced the number of bone marrow colony-forming unit granulocyte-macrophage (CFU-GM) in a time-dependent manner, while concomitantly stimulating high proliferative potential (HPP). In contrast, splenic CFU-GM and HPP were increased in a time- and dose-dependent manner. These data showed that IL-12 exerts *in vivo* hematopoietic effects that include the ability to mobilize progenitor cells to the peripheral circulation. In a clinical study in metastatic melanoma patients, Mortarini et al. [44] have reported that the SC administration of human recombinant IL-12 promoted the infiltration of neoplastic lesions by CD8+ T-cells with a memory phenotype.

The hallmark effect of IL-12 in immunity is its ability to stimulate the production of IFN-γ from NK cells, macrophages and T cells [6,10]. CXCL10 is a chemokine associated with T-cell responses and leukocyte migration, while IL-18 plays important roles in the development, homeostasis, and functions of CD4+ T cells, CD8+ T cells, and NK cells [45,46]. Additionally EPO was also analyzed as a systemic measure of rHuIL-12 activity based on its biological functions, other than erythropoiesis, that have been unraveled after finding EPO receptors on cells other than erythroid progenitors, such as polymorphonuclear leukocytes, megakaryocytes, and endothelial, myocardial, and neuronal cells [47-52]. EPO production was stimulated by rHuIL-12 in non-irradiated and irradiated NHP and mice demonstrating a potential central role in mediating the radiomitigation activity [6]. Thus induction and interrelationship between rHuIL-12 and a battery of these biological response parameters was studied in healthy human subjects.

The PD parameters in these studies were selected based on their relevance to immune-mediated effects of IL-12 that have been well documented in vitro [38,53,54], as well as in animal models [6] and clinical studies [19,55-61]. In our studies, IFN-γ, IL-18 and CXCL10 were found to be useful as downstream positive biological response parameters in support of the immunomodulatory activities of rHuIL-12. The IFN-γ response was consistently detected in both studies. The relatively delayed T_max_ together with the lack of endogenous IFN-γ indicate that this is a true biological IFN-γ response, which is known to be mediated via differentiation of TH1 cells. CXCL10 is another sensitive marker of induction of TH1-like innate immune activation manifested by IFN-γ, as well as directly stimulated by IL-12 [62,63]. Significantly higher levels of CXCL10 were detected in response to rHuIL-12, although results were confounded by variable endogenous levels. Based on the PD effects observed in these studies, we hypothesize that a single, low dose of exogenous rHuIL-12 is a trigger for multilineage hematopoietic stimulation and innate immune responses, which is unique and not achievable with currently used hematopoietic growth factors.

Currently rHuIL-12 is being developed as a radiation countermeasure under the provisions of FDA’s Animal Rule [28]. Nonclinical studies in rhesus monkeys have shown a similar pattern of transient hematological changes and immune responses with rHuIL-12 treatment in normal monkeys. Further, in the studies where monkeys were subjected to lethal total body irradiation, single, low doses of rHuIL-12 in the range of 50 to 500 ng/kg demonstrated an increased survival benefit in the absence of any supportive care (i.e., no antibiotics, fluids or blood products). The safe and well-tolerated human dose of 12 μg (171 ng/kg based on 70 kg human body weight) is within this efficacious range based on exposure parameters [64]. Based on these results, rHuIL-12 is being developed under the FDA Animal Rule approval pathway as a frontline therapy in humans to mitigate radiation-induced damage and increase the potential for survival in the event of a nuclear disaster or accident. We also have reported that low-dose, adjuvant IL-12 promotes multilineage hematopoietic recovery from cancer therapy-induced cytopenias, along with concomitant anti-tumor responses, in tumor-bearing mice [65]. Thus, results from these animal and human studies of rHuIL-12 also can be applied towards clinical trials in oncology, with the aim of reducing both toxicity and tachyphylactic effects that result from repeated high-dose regimens of IL-12, thereby augmenting antitumor effects.

## Conclusions

The observations from these studies indicate that a single low dose of rHuIl-12 administered subcutaneously can elicit hematological and immune-mediated effects without undue toxicity. The potent hematological and immune effects observed in our studies suggest that rHuIL-12, when dosed in a prudent manner, can be safely used to support the hematopoietic system and provide immunotherapeutic benefits in individuals exposed to lethal radiation, as well as in patients with myelosuppression resulting from either chemotherapy or radiation therapy.

## Methods

The clinical studies reported here have been approved by the Institutional Review Board (Independent IRB Inc., Fort Lauderdale FL 33323). All clinical investigations have been conducted according to the principles expressed in the *Declaration of Helsinki* and registered at ClinicalTrials.gov.

### Subjects

A total of 32 healthy subjects were enrolled in the first-in-human (FIH) study and 60 subjects were enrolled in the expansion study. Written informed consent (approved by the Independent IRB Inc., Ft. Lauderdale, FL) was obtained from all subjects prior to their participation in the study. Male and female healthy subjects between 18 and 45 years of age, with a body mass index > 19 and < 30 kg/m^2^, and normal ECG, physical examination, and laboratory results, and females with negative pregnancy test or not breastfeeding were enrolled in the study. Subjects with history of drug or alcohol addiction, food or drug allergies, or who had previously used IL-12 or were unable or unwilling to practice effective contraception were excluded. Use of ibuprofen was allowed to a total dosage of up to 2400 mg/day for flu-like symptoms experienced after study drug administration.

### Study design

The primary endpoint for both studies was safety and tolerability of rHuIL-12. Secondary endpoints were pharmacokinetic and pharmacodynamic profiles. The FIH study was conducted between 01 June 2011(first subject enrolled) and 20 March 2012 (last subject completed). The phase 1b expansion study was conducted between 05 August 2013 (first subject enrolled) and 03 November 2013 (last subject completed). Both studies were stopped following completion of the final study visit for the final subject. Subjects were enrolled by study staff. Randomization in both studies was accomplished using SAS®-generated randomization sequences (SAS® Institute Inc. Cary, North Carolina) that were assigned to subjects by an unblinded research staff member. All other research staff, subjects, care providers, and the sponsor were blinded to the treatment assignment.

In the double-blind FIH study, eligible subjects were enrolled in consecutive cohorts to receive either a single subcutaneous dose of rHuIL-12 (2, 5, 10, 20 μg) or placebo. Randomization for each cohort was 4:2 (rHuIL-12: placebo). Sentinel subjects, defined as the first subject in each cohort to receive rHuIL-12 and the first subject in each cohort to receive placebo were dosed at the beginning of each cohort. The starting 2 μg dose of rHuIL-12 was derived from allometric scaling of approximately one tenth of the NOAEL from rhesus monkey toxicology studies (Neumedicines, Inc., unpublished data), in accordance with FDA guidelines [31]; B), historical data from healthy subjects treated with a single dose (1 to 8 μg) of a different formulation of rHuIL-12 [10]; and C) data from patients with various types of cancer treated with rHuIL-12 at doses ranging from 0.003 to 1.25 μg/kg) [29,30]. Due to the occurrence of DLTs in the sentinel subject treated at the 20 μg dose level, an additional cohort of 6 subjects was randomized to rHuIL-12 at 15 μg or placebo, and due to the occurrence of a DLT in the final subject in that cohort, an additional cohort of 6 subjects was enrolled for treatment at 12 μg of rHuIL-12 or placebo. Thus, a total of 32 subjects were enrolled and randomized.

Subjects were admitted to the research facility at the Covance Clinical Research Unit (Madison, WI), 12 hours prior to study drug administration. Study drug was administered subcutaneously on day 1 following baseline assessments. Subjects resided in the research facility for 7 consecutive days and returned on days 11, 14 for interim visits and on day 28 for the close‒out visit. Blood samples for clinical chemistry and hematology assessments were collected at screening, baseline (day 1), and on days 2 to 7, 11, 14 and 28. PK samples were collected on day 1 (within 1 hour predose, and at 1, 2, 5, 8, 12, and 24 hours postdose) and daily on days 3 to 7, 11, and 14. PD samples were collected at screening, on day 1 (within 1 hour predose; at 5, 12, and 24 hours post dose), and daily on days 3 to 7, 11 and 14. Samples for immunogenicity testing were collected on day 1 (within 1 hour predose) and on day 28.

In the double-blind Phase 1b expansion study, eligible subjects were randomized at a 4:1 ratio to receive either a single subcutaneous 12 μg dose of rHuIL-12 or placebo and were dosed in three consecutive groups. Subjects were admitted to the research facility at Covance Clinical Research Unit (Madison, WI), 12 hours prior to study drug administration. Study drug was administered on day 1 following baseline assessments. Subjects resided in the research facility for 16 consecutive days and returned to the facility on day 28 and on day 45 (only those who consented per the amended protocol) for immunogenicity testing. The 16 day in-house stay facilitated thorough hematological analyses. Blood samples were collected for clinical laboratory investigations and immunogenicity tests from all 60 subjects at screening, daily on days 1 (within 1 hour pre-dose) to 16 and 28. Samples for PK and PD analyses were collected only from the subset of 40 subjects. PK samples were collected on day 1 (within 1 hour pre-dose and at 1, 2, 5, 8, 12 and 24 hr post dose); then daily on days 3 to 7. PD samples were collected on day -1, day 1 (within 1 hour predose and at 5, 12 and 24 hours post dose), then daily on days 3 to 7, 11, and 14. Peripheral blood samples for the flow cytometry analysis were collected on day-1, day 1 (within 1 hour pre-dose); then daily on days 2 to 7, 9, 11, and 14. Samples for immunogenicity testing were collected on day 1 (within 1 hour pre-dose) and on day 28 (all subjects) and day 45 (only for 34 subjects who consented to additional sampling).

### Analysis of safety

The safety and tolerability of rHuIL-12 were assessed by physical examination, vital sign measurements, laboratory tests, ECGs, and monitoring and reporting of adverse events (AEs). DLTs and AE severity were based on the FDA 2007 guidance on toxicity grading for healthy adult and adolescent volunteers enrolled in preventive vaccine clinical trials [31] with additional modifications by the FDA. For the FIH study only, this scale was modified with more stringent criteria for clinical and laboratory abnormalities. As only one dose of rHuIL-12 was given, subjects were not withdrawn from the study as a result of a DLT. All AEs were mapped to preferred terms and body systems using MedDRA version 15.1. Descriptive statistics were used for demographics, baseline characteristics; frequencies and percentages were used for adverse events by treatment assignment. In the expansion study, subjects from all 3 groups were combined to form the placebo and rHuIL-12 groups for comparison of endpoints. In both studies, the safety analysis population included all subjects who were randomized.

### Hematological assessment

A complete blood count (CBC) was done using a routine validated procedure at the local laboratory contracted by the Covance Clinical Research Unit (Madison, WI). Flow cytometric detection of immunophenotypes within the lymphocyte population, i.e. CD3^+^, CD4^+^, and CD8^+^ lymphocytes, was done at the Covance Immunotoxicology Laboratory (Madison,WI) using a 4-color Multitest Kit (BD Biosciences; San Diego, CA; Cat #3400491).

**Figure 6:**
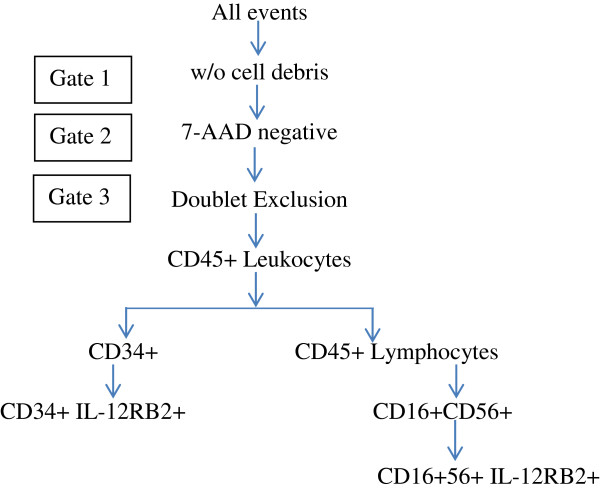
Gating strategy for flow cytometry in Phase 1 b expansion study.

Additionally, NK cells and circulating hematopoietic progenitors were measured by flow cytometry. NK cells were defined to be the CD45^+^CD16^+^CD56^+^ lymphocyte population, and circulating hematopoietic progenitors were defined to be CD45^+^CD34^+^ leukocytes. NK cell gating was performed in two steps: firstly, lymphocytes were gated using CD45 and side scatter, followed by gating on CD16^+^CD56^+^ double-positive population. CD3 negativity was not used for NK cell gating. Dead cells were eliminated based on 7AAD positivity. IL-12Rβ2 positivity was further determined on the NK and CD34^+^ cells. Fluorescently labeled antibodies against 7-AAD, CD45, CD56, CD16, and CD34, were purchased from BD Biosciences (San Jose, CA; Cat. #559925, 641399, 557699, 557744, and 348057, respectively) and the anti-IL-12 Rβ2 antibody, was purchased from R&D Systems (Minneapolis, MN; Cat. #FAB1959A). Entire gating strategy is delineated in the Figure 6 below:

### Bioanalytical assays

All enzyme linked immunosorbent assay (ELISA) methods were validated in human K2 EDTA plasma using commercially available kits and antibodies. These assays were performed at Intertek Pharmaceutical Services, San Diego, CA. Assay systems and LLOQ were as follows: rHuIL-12 - Quantikine® HS Human IL-12 High Sensitivity ELISA Kit, (R&D Systems; Minneapolis, MN; Cat. #HS120, SS120, or PHS120) and LLOQ = 3.75 pg/mL; IFN-γ. Human Interferon-γ (IFN-γ) ELISA MAX™ Deluxe Sets, (BioLegend Inc.; San Diego, CA; Cat. #430106) and LLOQ = 30.00 pg/mL; EPO - Quantikine® IVD® Erythropoietin ELISA kit, (R&D Systems; Cat. #DEP00) and LLOQ = 2.00 mIU/mL; IL-18 - Human IL-18 ELISA Kit, MBL (Japan), distributed by R&D Systems (Cat #7620) and LLOQ = 25.60 pg/mL; CXCL10 - Quantikine® Human CXCL10 Immunoassay ELISA Kit, (R&D Systems; Cat. #DIP100) and LLOQ = 12.00 pg/mL. Immunogenicity testing (anti rHuIL-12 antibodies) used a tiered cut-point titration method validated at Intertek Pharmaceutical Services, utilizing anti-human IL-12 (p70) antibody (BioLegend Inc.; Cat. #511002); LLOQ was not applicable.

### Pharmacokinetic and pharmacodynamic analyses

Plasma PK analyses for rHuIL-12, and PD analyses for IFN-γ, EPO, IL-18 and CXCL10 were performed by ProPharma Services LLC, Superior CO using non-compartmental methods within Phoenix™ WinNonlin® Version 6.3 or higher (Pharsight Corporation; Mountain View, California). Nominal dose and actual weight and sampling times were used. Summary statistics were prepared with WinNonlin and Microsoft® Excel 2007 (Seattle, WA), while mean and individual concentration graphs were prepared with Prism™ Version 5.0 (GraphPad Software, Inc.; La Jolla, CA). Descriptive statistics were provided for PK and PD by treatment and by gender. Group and gender comparisons of means for C_max_, AUC_0-t_ and AUC_0-∞_ values were conducted via a *t*-test with log-transformed data using MedCalc® version 12.2.1.0 (Mariakerke, Belgium).

### Flow cytometry analysis

Fluorescence-activated cell sorting (FACS) DIVA® software Version 6.1.3 (BD Biosciences; San Diego, CA) was used for data acquisition and analysis at the Covance Immunotoxicology Laboratory (Madison,WI) and Neumedicines Inc., Pasadena CA.

## Abbreviations

rHuIL-12: Recombinant human interleukin 12; FIH: First-in-human; AE: Adverse event; IL-18: Interleukin-18; NK: Natural killer; TH: T-helper; IFN-γ: Interferon gamma; MCM: Medical countermeasure; DLT: Dose-limiting toxicity; HSARS: Hematopoietic syndrome of the acute radiation syndrome; NHP: Non-human primate; MTD: Maximum tolerated dose; PK: Pharmacokinetic; CXCL10: C-X-C motif chemokine 10; PD: Pharmacodynamic; AUC: Area-under-the-curve; EPO: Erythropoietin.

## Competing interests

Financial Competing Interests: MSG, VV, JT, ST HK, CEL and ZGP are employees of and own equity of $10,000 or more in Neumedicines, Inc. LAB is the president and CEO of Neumedicines, Inc. and owns equity of $10,000 or more in Neumedicines, Inc. LAB holds patent 7,939,058 relevant to this work. NS is an employee of Covance, Inc. and received monies for the services rendered.

## Authors’ contributions

MSG, VV, JT, ST HK, CEL, ZGP, NS and LAB are credited for: Substantial contributions to the conception or design of the work; or the acquisition, analysis, or interpretation of data for the work; and Drafting the work or revising it critically for important intellectual content; and Final approval of the version to be published; and Agreement to be accountable for all aspects of the work in ensuring that questions related to the accuracy or integrity of any part of the work are appropriately investigated and resolved.
